# Dissociated Effects of Age and Recent Troubling Experiences on Nightmares, Threats and Negative Emotions in Dreams

**DOI:** 10.3389/fpsyt.2022.770380

**Published:** 2022-03-03

**Authors:** Kheana Barbeau, Alexandre Lafrenière, Hanae Ben Massaoud, Emma Campbell, Joseph De Koninck

**Affiliations:** ^1^School of Psychology, University of Ottawa, Ottawa, ON, Canada; ^2^Department of Psychology, Université de Montréal, Montreal, QC, Canada

**Keywords:** dreams, troubling experiences, nightmares, emotions, dream formation, neurocognitive model of nightmares, threat simulation theory, continuity hypothesis

## Abstract

Several studies have highlighted associations between adverse life events and the dysphoric character of dream experiences. This degree of continuity between waking-life and dream content seems partly attributed to the emotional and personal attachment linked to the incorporated waking experiences. Numerous changes in the processing of emotion-related stimuli are also reported across different human developmental stages. Therefore, we were interested in testing whether age would modulate the impact of recent troubling experiences on dream characteristics. Two hundred sixty participants, evenly distributed in five developmental stages, matched for gender and their exposure to a troubling experience, were selected from a large sample collected for a previous normative study of dreams of Canadians. Participants completed a dream questionnaire from which independent judges subsequently scored the dreams. We observed no interactions between the experience of troubling events and age. However, individuals who experienced a recent troubling event reported a higher frequency of nightmares and their dreams were more emotionally negative. Participants who experienced a moderately severe troubling event were also more likely to experience a dream whose maximal threat severity was of moderate intensity. Adolescents and young adults had dreams with a higher level of oneiric threats compared to older adults (>40 years old). Young adults also reported a higher frequency of nightmares compared to older adults. Our findings have implications for modern dream theories. They also suggest that dysphoric dreams might serve as potential proxies of mental health status and developmental stages. Future studies are now needed to explore the implications of these findings for psychological adaptation.

## Introduction

There is evidence for a certain degree of continuity between waking-life and dream content. It is postulated that dream features reflect waking-life experiences and/or that dreams impact psychological adaptation. The former concern dream formation theories and the latter dream function theories. Several specific proposals on how this occurs have been formulated and fall under the umbrella of what is best known as the continuity hypothesis (CH) of dreams [see an overview by (1)]. Research so far suggests that the reflection of waking life in dreams is mostly selective, distorted and that features, such as reading and writing (i.e., mundane tasks), very seldomly find their way into dreams (2). More specifically, continuity between dream content and waking life was observed within complex individual characteristics, such as personality (3, 4), physical health (5), social roles (6) and individual concerns (7, 8). It was also found that changes in psychological well-being are correlated with similar changes in emotional dream content (9) and significant life experiences such as pregnancy can be well represented with distortions in dreams (10). Schredl (11) has proposed a mathematical model of families of influencing factors and their interactions that mediate incorporation into dreams. Of overall interest is the observation that, once the proper physiological substrate and cognitive capacity are achieved, dream construction prioritizes emotional waking-life experiences and concerns with a negative bias (12, 13).

Along the same line, stressful life experiences have been shown to be preferentially incorporated into dreams (14, 15). There is strong evidence of this effect in those who have experienced a traumatic or severe adverse life event and subsequently suffered from post-traumatic nightmares (16, 17). Indeed, several studies have found a relationship between traumatic experiences during waking-life and the subsequent development of dysphoric dreams and chronic nightmares (18–21). Interestingly, current research examining the impact of the 2019 Coronavirus (COVID-19) has reported increased nightmares, anxiety, and threats in dreams (22–25). Mounting evidence supports the idea that personally and emotionally difficult waking experiences, which may vary in temporal proximity, can have a long-lasting impact on the everyday oneiric experience. For example, a history of severe childhood trauma in undergraduate women was associated with more frequent disturbing dreams, higher nightmare distress and augmented psychopathology (26). Similarly, a recent study reported that the dreams of war prisoners during World War II had long-lasting incorporations of war-related themes, threatening elements, and negative emotions (27). Adverse life events may thus be overrepresented in dreams because they continue to represent real-life concerns and are of personal significance (28). From a dream construction perspective, these two elements have been identified as factors that affect the degree of continuity between waking-life and dream content (28).

According to the Threat Simulation Theory (TST), dreaming is thought to have evolved as an adaptive mechanism to simulate oneiric threats drawn from waking-life experiences, and the repetition of these threats in dreams were thought to improve survival skills (29). Although the TST was proposed to explain the evolutionary function of dreaming in our previous human ancestors, the theory has been investigated in modern life and societies. For instance, those who have a more active threat simulation system (TSS), such as those who have endured a trauma, have been shown to report more severe dream threats (30). Other studies have also found that the frequency and severity of waking-life threatening events are associated with the severity and frequency of dream threats (31, 32). Additionally, pre-sleep negative emotions were shown to be associated with threats in dreams (31), suggesting that waking life emotional experiences influence the menacing characteristics of dreams. In sum, the CH and the TST offer an explanation for the incorporation of waking-life events into dreams. The TST goes further by postulating that dreams served an evolutionary biological function in ancestral times (33). This phylogenetic function is impossible to directly test in modern days, as recognized by the TST (33, 34). However, what can be tested is whether threatening dreams form following adverse life experiences.

Nightmares are defined as elaborated and highly dysphoric dreams whose content is threatening and accompanied by intense negative emotions (35). While several recent models of nightmare formation have been developed from a psychological (36) to a neurochemical (37) perspective, a predominant view of interest for the current study stems from the neurocognitive model of nightmares [NMN; (38)]. It proposes that nightmares result from a dysfunction in a network of affective processes that typically serve the adaptive function of fear-memory extinction during dreaming (38). Thus, “normal dreaming” would facilitate fear-memory extinction through memory-element activation (e.g., deconstruction of memories into isolated elements); memory-element recombination (e.g., new elements are combined with isolated memory-specific elements to be inconsistent with real memories and offer novelty for enhanced emotional processing); and emotional expression [e.g., attentional focus on dream imagery to downregulate negative emotional arousal; (38)]. The link between adverse life experiences and nightmares is thus explained by a disruption in these dream processes caused by the accumulation of stress and negative affect from these life events (i.e., high affective load). This would ultimately lead to more incorporation of distressing memories into dreams. The relationship between adverse life events and nightmares may also be modulated by age. For example, it was reported that early life exposure to negative adverse events is associated with later nightmares and dysphoric dream experiences (26, 39–41). Moreover, the frequency of nightmares also changes with age. The direction of this change remains, however, to be clarified, as some cross-sectional studies observed with advancing age an increase (39, 42), a decrease (43–46), or no changes (47) for the frequency of nightmares. Nevertheless, more studies seem to point in the direction of a general decline in nightmares frequency with advancement in age. Congruently, in a study investigating non-trauma exposed adults, it was shown that the younger the participants were, the higher were the frequency of dream threats (48). To date, our understanding of the potential age-related effects on dysphoric characteristics of dreams in those who experienced a negative adverse event is limited and has not been investigated in an ontogenetic manner.

Although there are no theories to explain these age-related differences, neurodevelopmental changes associated with brain maturation and aging may explain differences in perceptual processing and memory storage of negative stimuli. For instance, compared to older adults, adolescents demonstrate greater activity in areas of the brain responsible for processing emotions, specifically areas implicated in the fear response (e.g., amygdala), when viewing negative stimuli (49–51). These findings suggest that adolescents are more emotionally reactive to negative stimuli than older adults. Additionally, older adults demonstrate lower activation in these regions (52, 53) or a stronger coupled activation with prefrontal areas of the brain compared to younger adults when viewing negative stimuli (54), signifying better emotion regulation and less negative emotionality. Adolescents and young adults may demonstrate higher fear responses when viewing negative stimuli because their limbic structures, such as their amygdala, are more developed than prefrontal structures during this life stage, and thus functional connectivity between these regions in the brain are weaker, limiting the capacity for emotion regulation (55). These differences in fear responses when viewing negative stimuli are also associated with biases in memory retrieval. Indeed, younger adults are more likely to recall negative stimuli (56), while older adults are more likely to recall positive stimuli (57–59). Taken together, these findings suggest that neurodevelopmental processes may underlie age-related differences in experiencing threatening and dysphoric elements in dreams and nightmares. More specifically, adolescents and younger adults may be more likely to perceive situations or elements of their environment as more threatening and be more likely to recall these events, leading to higher incorporation of these memories into their dreams. Furthermore, some of their affective processes are limited, such as emotion regulation, due to brain immaturity in frontal regions, which may impede the facilitation of fear-extinction processes that naturally occur during “normal dreaming,” resulting in fear-enhancing dreams (i.e., nightmares).

Although associations have been found between adverse life events and the disturbing and threatening nature of dreams, it remains unclear if those who have experienced a common (e.g., a death, a separation, interpersonal difficulties, an accident) and recent (i.e., within the past year) troubling personal event would report more nightmares, have a higher level of threat and negative emotions in their dreams. Furthermore, the degree to which there is continuity between the severity of recent adverse events and the threat severity of dreams remains unclear in a community-based population. Finally, despite observations of age-related differences in negative dream characteristics, studies have yet to take a developmental approach.

The main objective of the current study was thus to investigate the potential interaction between age and the experience of a recent troubling event on the dysphoric characteristics of dreams. In terms of dream characteristics, we were interested in the frequency of nightmares, the “threatening tone” of dreams, and the level of positive and negative dream emotions. We selected these variables as they measure the dysphoric experience of dreams at the level of monthly occurrence, actual threatening content, and emotions, respectively. Finally, our last objective was to assess which relevant dream theory (i.e., CH, TST, NMN) would best explain our findings.

## Hypotheses and Predictions

The CH (11) and TST (33) posit a certain degree of continuity between waking negative experiences and subsequent dream content. Thus, we predicted that, compared to participants who did not experience a recent troubling event, those who did will have more dysphoric dreams, which will manifest through a higher frequency of nightmares, higher level of oneiric threat, higher negative dream emotions and lower positive emotions. We also predicted that the severity of troubling events will be associated with the severity of dream threats: minor troubling experiences will be associated with minor oneiric threats, moderately troubling experiences will be associated with moderate oneiric threats, and severe troubling experiences will be associated with severe oneiric threats. It should be noted that, for the current study, the predictions drawn from the CH and TST cannot be differentiated.

The NMN (21, 38) proposes that the accumulation of stressful and negative emotional experiences (i.e., affect load) during wakefulness can entail the experience of disturbing dreams. Consequently, we predict that, compared to participants who did not experience a recent troubling event, those who did will have a higher frequency of nightmares and a higher level of negative emotions in their dreams. Of note, the NMN postulates that dreams regulate fear-related emotions by recombining fear-memories with non-fearful mnemonic elements into dreams. Therefore, we propose that this mechanism could be observed at the level of dream emotions, where the positive and negative emotions would tend toward a “relative” equilibrium in their intensities to regulate the impact of negative dream emotions. Thus, compared to individuals who did not experience a recent troubling event, those who did should have either a comparable or higher level of positive emotions in their dreams to match or outmatch their expected higher level of negative emotions. To the best of our knowledge, this prediction has never been tested before.

The neurodevelopmental paradigm highlights multiple changes in the perception and processing of emotion-related stimuli from adolescence to older ages (60–63), and the dream formation literature points to a link between waking-life and dream experiences (11, 33, 38). Moreover, age-related differences have been found in the frequency of nightmares (43–47) and oneiric threats (48). Collectively, these led to the prediction that adolescents and younger adults will report a higher frequency of nightmares and level of dream threats compared to middle-aged and older adults. This effect will be magnified in those who had a recent troubling experience.

## Method

### Participants and Protocol Overview

Two hundred sixty participants were selected from a large sample collected between 2004 and 2017 for a normative study of the dreams of Canadians (64, 65). Therefore, it was completed before the COVID 19 pandemic. Participants were between the ages of 12–90 years old (Mean = 38.0, *SD* = 21.3) and were devised into five age groups according to key developmental stages: adolescence (12–17 years old), early adulthood (18–24 years old), adulthood (25–39 years old), middle adulthood (40–64 years old), and late adulthood (65 years old and older). There were 52 participants who were matched for gender and exposure to a recent troubling experience within the past year in each age group (*N* = 260, men = 127; women 133; exposed = 129; non-exposed = 129). Of those who experienced a recent troubling event over the past year, most experiences were categorized as psychological, social, or economic adverse events (*n* = 79, 61%), followed by minor events (*n* = 32, 25%), deadly (*n* = 13, 10%), and physical (*n* = 5, 4%). All age groups included 52 participants who were matched for gender (men = 127; women = 133) and their exposure to a troubling experience within the past year (non-exposed = 131; exposed = 129). Chi-square tests confirmed that the proportion of gender (*χ*^2^_(4)_ = 0.25, *p* = 0.99) and exposure to a troubling experience (*χ*^2^_(4)_ = 3.45, *p* = 0.49) were similar across age groups. The study was approved by the Research Ethics Boards (REB) at the University of Ottawa.

Participants were recruited using the following approaches: through personal contacts at school boards, advertisements displayed at a Canadian university, advertisements on social media, at public presentations and conferences, at retiree associations, and word of mouth. All participants were unaware of the purposes of the study and provided written consent. After obtaining participant's consent, they were instructed to complete a dream questionnaire using pen and paper until at least two dreams were reported, for a maximum period of ten days. The dream questionnaire (DQ) included several sections, some of them described below are based on existing questionnaires, most notably on dream recall and nightmare frequency. No new validation procedure was applied. It was developed for the Normative Study of the dreams of Canadians that has led to several publications [see (64–66)]. The first section contained the consent form and instructions regarding how to fill out the questionnaire. The other sections contained sociodemographic questions and subsections about the characteristics of their dreams. The subsections of the questionnaire used in the current study are described below.

### Measures

#### Sociodemographics and Troubling Events

The DQ included a sociodemographic questionnaire in which the participants were asked to provide a detailed account regarding general information about them (e.g., age, gender, marital status, profession, education). Participants also had to report whether they had experienced any troubling events (e.g., a death, a separation, interpersonal difficulties, an accident) over the past year and, if so, to describe them.

#### Frequency of Nightmares

Next, the questionnaire required the participants to self-report their monthly frequency of nightmares, similar to the one for dream recall frequency, by checking one of the following categories: Less than once a month, Approximately once a month, Approximately once every two weeks, Approximately once a week, Many times a week, and Almost every night. These categories were recoded from 1 (i.e., less than once a month) to 6 (i.e., almost every night) and were used to conceptualize the frequency of nightmares.

#### Dream Reports and Emotions

Following this, participants filled out the morning section of the DQ and describe the narrative of their dream as soon as they wake up in the morning. The mean word count of dream narratives was 145.18 (range: 50–531; *SD* = 84.68). After describing the dream, they were instructed to assess the degree of joy, happiness, apprehension, anger, sadness, confusion, fear and anxiety [the dream emotion categories used by Hall et al. (67)] experienced in their dream on a four-point Likert scale (1 = not at all, 2 = a little, 3 = moderate, 4 = a lot). Participants' ratings of their dream emotions were recoded to range between 0 and 3, such that the level “not at all” started with a value of “0” to denote the absence of emotional experience. Then, the ratings of apprehension, anger, sadness, fear, and anxiety were averaged to form a mean intensity measure of the dream's negative emotions. The ratings of joy and happiness were averaged to produce the mean level of the dream's positive emotions. Cronbach's alpha of the positive and negative emotion scores were 0.91, 0.67, respectively.

#### Dream Threat Scale and Threat Severity Scale

For evaluating participant's threats in dreams, the third section of the Dream Threat Scale was used (68), which relates to the severity of the threats for the self. As in previous studies (31, 32), the definitions of this subscale (Life-threatening event; socially, psychologically or financially severe threat; physically severe threat; and a minor threat) were used for the identification of threats in the DQ. The level of threat severity was further rated on a four-point scale (0 = not threatening, 1 = somewhat threatening, 2 = moderately threatening, 3 = highly threatening). This allowed the assessment of the dream threats severity regardless of its qualitative nature. A similar scale was used in a previous study investigating aggressions in dreams of soldiers, gamers, and control participants (69). Given the relationship between the frequency of dream components and the reports length (64, 65), the frequency of dream threats were divided by the word count for the purpose of the analyses. Moreover, the most severe dream threat was selected as the measure of threat severity. A global measure of the dream's “threatening tone” was computed by conducting a z-transformation of the threat severity and frequency scores. After this transformation, both scores were averaged to create a threats composite score with higher values reflecting more severe and frequent dream threats. Cronbach's alpha was 0.81 for the threat composite score.

#### Scoring Procedure of Threat Characteristics of Adverse Events and Dreams

All participant's dreams were coded for the presence of oneiric threats. The dreams were coded by two independent judges who received instructions on how to identify the threats in the reports and how to evaluate their severity. The judges were trained on reports from other sources before starting the scoring of the participant's dreams. The threatening components in dreams were considered as such on the basis that both judges had identified the same elements as menacing using the aforementioned definitions in the DTS. If both judges disagreed on the threatening nature of an event, the scoring of this event was discussed until they reached an agreement. The inter-rater reliability was evaluated for the level of threats severity using the intraclass correlation coefficient's (ICC) average measure parametrized for an absolute agreement definition. All ICC values ranged from 0.90 to 0.94, suggesting excellent reliability between the judge's scoring. The same procedure was undertaken for the scoring of the troubling events experienced within the past year. The ICC for the severity level of threats was 0.78, suggesting good reliability. Following the scoring phase, the first dream containing a minimum of 50 words and a maximum of 550 words was retained for analyses. Five participants (1.92%) were excluded from analyses as they reported dreams with an insufficient word count. Therefore, 255 participants were retained and included in the main analyses.

### Data Analytical Plan

For the main analysis, a series of 2 (recent troubling event: yes or no) by 5 (age group: 12–17 years old; 18–24 years old; 25–39 years old; 40–64 years old; and 65 years or older) between-subjects ANOVAs were conducted to examine the relationship between experiences of recent troubling events and age on the threatening tone of dreams, frequency of nightmares, and emotional levels of dreams (positive and negative). Bonferroni adjustments were applied to *post-hoc* tests. To examine whether the severity of a recent troubling event is associated with severity of oneiric threats, a 3 by 3 (somewhat threatening, moderately threatening, and highly threatening) chi-square test of independence was conducted in those who experienced a recent troubling event and had dreams that contained threatening events. Only those with a threatening dream were included in this analysis as we were interested in whether the severity of troubling events during wakefulness was associated with the maximal severity of threatening events in dreams. This allowed us to test the degree of continuity between the severity of threatening experiences during wakefulness and dreaming. Bonferroni corrections were applied to *post-hoc* comparisons (0.05/3 = 0.0166). All statistical analyses were conducted using IBM SPSS v27 (SPSS, 2021) and figures were created using GraphPad Prism v9 (2021) for Windows.

For a between-subjects ANOVA with two factors, an a priori power analysis using G^*^Power (70) recommended a sample size of 223 participants for detecting a medium effect size with power of 0.70 and an alpha of 0.05, thus our goal was to reach this sample size. For a two-tailed 3 by 3 Chi-square test of independence, an a priori power analysis using G^*^Power (70) recommended a sample size of 108 participants for detecting a medium effect size with a power of 0.70 and an alpha of 0.05.

## Results

### Preliminary Analyses

Regarding data cleaning, missing data were not imputed; therefore, participants with missing data were omitted from certain ANOVA analyses (*n* = 4 excluded; three from the exposed group and one from the non-exposed group; *N* = 255 for the final sample). Some participants were missing data due to variations in questionnaire packages over the duration of the data collection period for the normative study and thus did not have the opportunity to self-report on all outcomes of interest in the current study. Univariate outliers were winsorized. There were no multivariate outliers. Assumptions for the 2 (recent troubling event) by 5 (age group) between-subjects ANOVAs for the main analyses for normality and homogeneity of variance were examined through scatterplots and tests of homogeneity of variance. The assumption for homogeneity of variance was met across all outcomes. There were minor violations of normality; however, transformations were not applied considering that analysis of variance tests are robust to non-normality (71), especially in cases where group variances are similar (72). Means and standard deviations for all outcomes of interest are displayed in Table 1.

**Table 1:** Descriptive statistics stratified by adverse event exposure within the past year (AE) and age group.

**Group**	**12–17 AE**	**12–17 No AE**	**18–24 AE**	**18–24 No AE**	**25–39 AE**	**25–39 No AE**	**40–64 AE**	**40–64 No AE**	**65 + AE**	**65+ No AE**
Mean (*SD*) severity of AE	2.31 (0.65)	–	2.00 (0.82)	–	2.25 (0.79)	–	1.93 (0.98)	–	2.10 (0.94)	–
Mean (*SD*) nightmare frequency	2.10 (1.19)	1.86 (1.00)	2.62 (1.56)	2.13 (1.00)	2.13 (1.51)	1.77 (1.14)	1.48 (0.94)	1.58 (0.99)	1.69 (1.11)	1.05 (0.23)
Mean (*SD*) positive emotions in dreams	1.18 (1.23)	1.02 (1.02)	0.74 (0.96)	0.78 (0.89)	0.63 (0.77)	0.90 (1.12)	1.04 (1.04)	0.60 (0.90)	1.18 (1.17)	0.74 (1.09)
Mean (*SD*) negative emotions in dreams	1.11 (0.57)	0.91 (0.64)	1.23 (0.61)	0.96 (0.65)	0.91 (0.81)	0.98 (0.80)	0.91 (0.74)	0.66 (0.68)	0.94 (0.92)	0.53 (0.60)
Mean (*SD*) threatening tone of dreams	0.28 (0.91)	0.64 (1.13)	0.26 (0.85)	0.21 (0.74)	0.15 (0.79)	−0.14 (0.77)	−0.43 (0.84)	−0.30 (0.75)	−0.20 (0.83)	−0.47 (0.76)
Mean (*SD*) max severity of threats in dreams	2.11 (0.76)	2.29 (0.75)	2.00 (0.91)	1.92 (0.74)	1.95 (0.89)	1.88 (0.81)	1.92 (0.86)	1.47 (0.74)	1.76 (0.83)	2.00 (0.87)

### Main Analyses

#### Differences in the Threatening Tone of Dreams: Threats Composite Score

**Figure 1:**
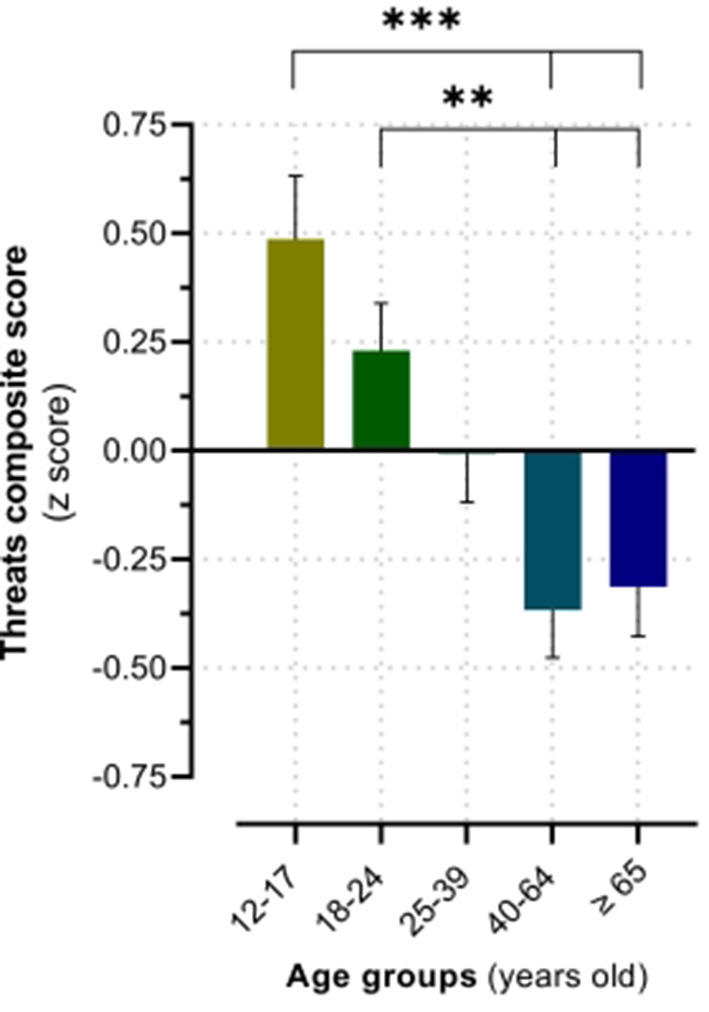
Age group differences in the threatening tone of dreams. The values are expressed as means ± SEM, *N* = 255. **p < 0.01, ***p < 0.001.

A 2 (recent troubling event) by 5 (age group) ANOVA demonstrated a non-significant main effect of recent troubling event, *F*_(1, 245)_ = 0.55, *p* = 0.815, $\eta_{\text{p}}^{2}$ = 0.000, a significant main effect of age group, *F*_(4, 245)_ = 8.94, *p* = <0.001, $\eta_{\text{p}}^{2}$ =0.127, and a non-significant interaction between recent troubling event and age group, *F*_(4, 245)_ = 1.31, *p* = 0.269, $\eta_{\text{p}}^{2}$ = 0.021, for the composite score of oneiric threats. As shown in Figure 1, pairwise comparisons revealed that 12–17 year old's significantly had more severe and frequent oneiric threats compared those who were 40 years old or older (40–64 years old: *p* = <0.001; 65 years old or older: *p* = <0.001). Similarly, 18–24 year old's had significantly more severe and frequent oneiric threats compared those who were 40 years old or older (40–64 years old: *p* = 0.004; 65 years old or older: *p* = 0.009). No other age differences emerged.

#### Differences in Frequency of Nightmares

**Figure 2:**
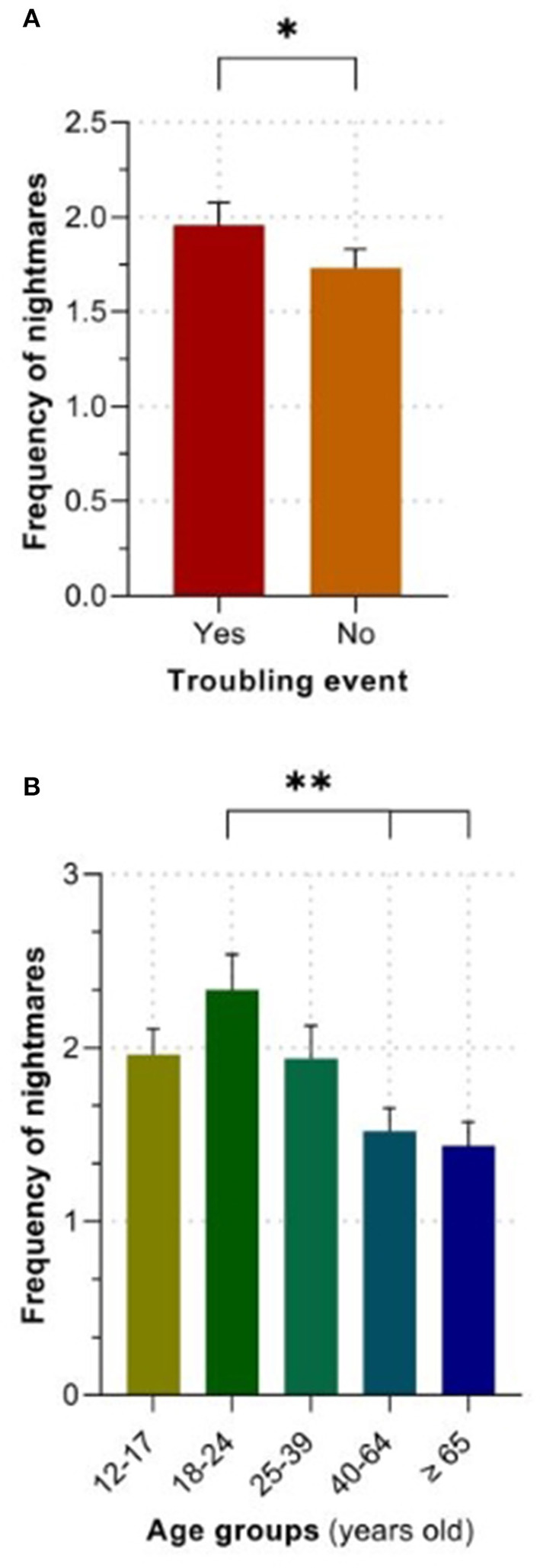
Group differences in the frequency of nightmares. **(A)**. Main effect of the experience of troubling events over the past year. **(B)**. Main effect of age. The values are expressed as means ± SEM, *N* = 250. *p < 0.05; **p < 0.01; ***p < 0.01.

A 2 (recent troubling event) by 5 (age group) ANOVA demonstrated a significant main effect of recent troubling event, *F*_(1, 240)_ = 4.74, *p* = 0.030, $\eta_{\text{p}}^{2}$ = 0.019, a significant main effect of age group, *F*_(4, 240)_ = 5.66, *p* = <0.001, $\eta_{\text{p}}^{2}$ = 0.086, and a non-significant interaction between recent troubling event and age group, *F*_(4, 240)_ = 0.67, *p* = 0.614, $\eta_{\text{p}}^{2}$ = 0.011, for the monthly frequency of nightmares. Pairwise comparisons revealed that those who experienced a recent troubling event reported a higher frequency of nightmares compared to those who did not (*p* = 0.030; see Figure 2A). Additionally, as depicted in Figures 2B, 18–24 year old's had significantly more nightmares compared to those who were 40 years old or older (40–64 years old: *p* = 0.005; 65 years old or older: *p* = 0.002). No other age differences emerged.

#### Differences in Positive Dream Emotions

**Figure 3:**
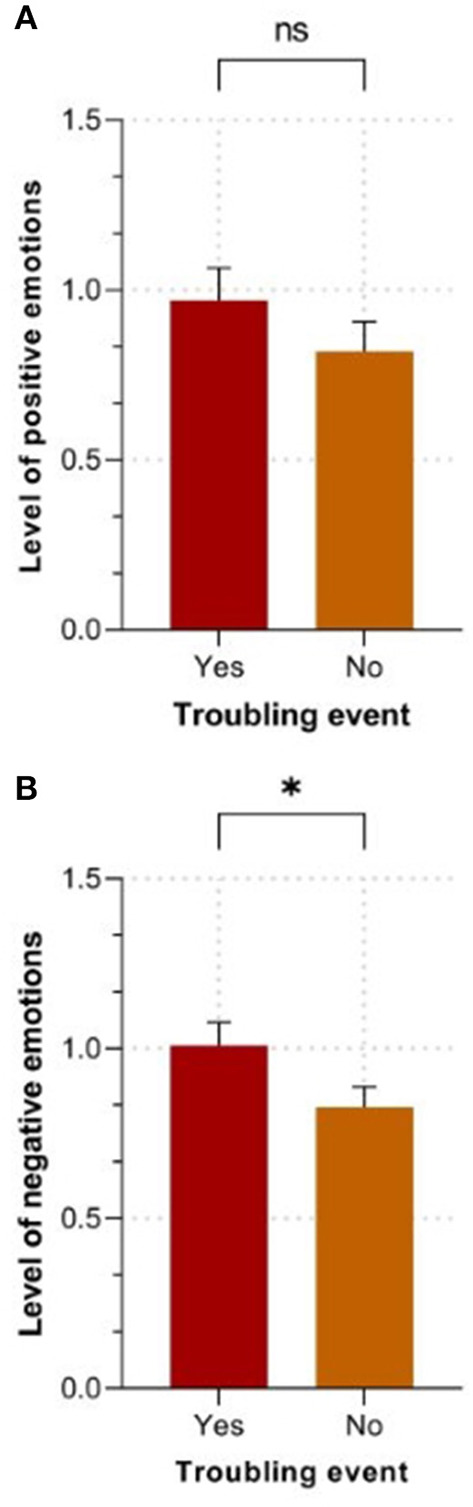
Main effect of the experience of a recent troubling event on emotion levels in dreams. **(A)**. Level of positive emotions. **(B)**. Level of negative emotions. The values are expressed as means ± SEM, *N* = 253. *p < 0.05, ns = *p* > 0.05.

A 2 (recent troubling event) by 5 (age group) ANOVA demonstrated a non-significant main effect of recent troubling event, *F*_(1, 243)_ = 1.22, *p* = 0.270, $\eta_{\text{p}}^{2}$ =0.005 (see Figure 3A), a non-significant main effect of age group, *F*_(4, 243)_ = 1.00, *p* = 0.407, $\eta_{\text{p}}^{2}$ = 0.016, and a non-significant interaction between recent troubling event and age group, *F*_(4, 243)_ = 1.12, *p* = 0.348, $\eta_{\text{p}}^{2}$ = 0.018, for positive emotions felt in dreams. Pairwise comparisons were not examined due to non-significant main effects.

#### Differences in Negative Dream Emotions

A 2 (recent troubling event) by 5 (age group) ANOVA demonstrated a significant main effect of recent troubling event, *F*_(1, 243)_ = 5.55, *p* = 0.019, $\eta_{\text{p}}^{2}$ = 0.022, a non-significant main effect of age group, *F*_(4, 243)_ = 2.23, *p* = 0.067, $\eta_{\text{p}}^{2}$ = 0.035, and a non-significant interaction between recent troubling event and age group, *F*_(4, 243)_ = 0.71, *p* = 0.588, $\eta_{\text{p}}^{2}$ = 0.011, for negative emotions experienced in dreams. Pairwise comparisons revealed that those who experienced a recent troubling event had a significantly higher level of negative emotions in their dreams than those who did not have a recent troubling experience, *p* = 0.046 (see Figure 3B).

#### Associations Between Severity of a Recent Troubling Event and Severity of Oneiric Threats

**Figure 4:**
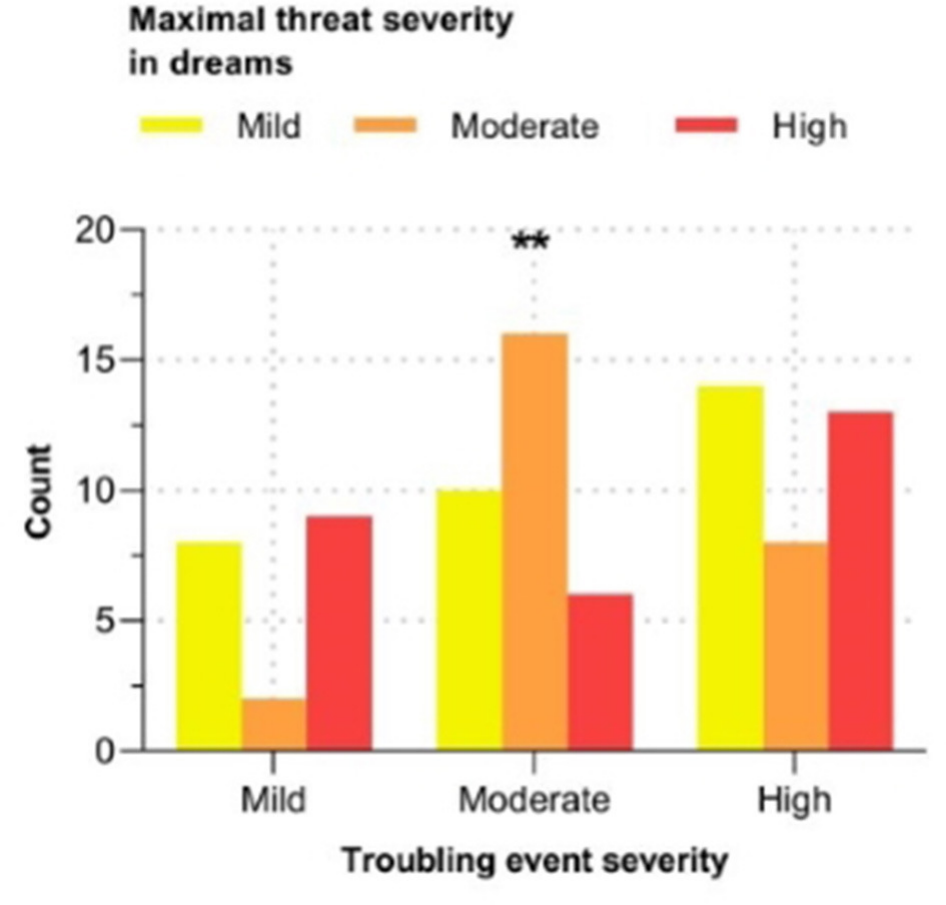
Associations between the severity of a recent troubling event and severity of oneiric threats. The values are expressed as count. *N* = 125. **p < 0.01.

In those who recently experienced a troubling event and had a threatening dream (*N* = 125), a 3 (severity of troubling event) by 3 (severity of oneiric threat) chi-square test of independence was conducted to examine the association between severity of the event and maximal severity of threats in dreams. This analysis revealed that the severity of a recent troubling event and severity of threats in dreams are significantly associated, *X*^2^_(4)_ = 11.09, *p* = 0.026. *Post-hoc* comparisons demonstrated that recently experiencing a moderately threatening event during waking was significantly associated with moderate severity for the maximal threat in dreams, *p* = 0.002 (see Figure 4).

## Discussion

In the context of recent dream formation theories, the objective of the current study was to examine whether common and recent adverse events were related to dysphoric dream characteristics. Given that the perception and processing of emotion-related stimuli undergo several changes from adolescence to older ages (60–63), we were also interested in examining whether age would modulate the influence of recent adverse experiences on subsequent dream characteristics. Aligned with our hypotheses, our results suggest that having a recent troubling experience is associated with reporting more nightmares monthly and experiencing a higher level of negative dream emotions. However, these adverse experiences were not related to the experience of dreams with a higher threatening tone. Additionally, we only observed a small degree of continuity between the severity of troubling events experienced within the last year and severity of threats in dreams, as this relationship was only present when the adverse event and dream threat were of moderate intensity. Furthermore, adolescents and younger adults had a higher level of threats in dreams compared to older age groups. Younger adults also self-reported a higher frequency of nightmares than adults older than 40 years old. Contrary to our hypotheses and predictions, no interactions between the experience of troubling personal events and age group were observed for any dream characteristic. The absence of significant interactions in the presence of both factors' significant main effects thus suggests a dissociation between these variables, which show specific and different impacts on the dreaming experience. Results are more thoroughly discussed and interpreted through the lens of three relevant modern dream theories in the following sections.

### Effects of Recent Troubling Events on Dream Characteristics

The NMN (21, 38) postulates that a higher level of affect load during wakefulness could precipitate the occurrence of disturbing dreams. One could safely speculate that experiencing a troubling event within the previous year may manifest through dysphoric emotions and stress levels experienced in daily life. Thus, as predicted by the NMN, we observed that individuals who experienced a recent troubling event self-reported a higher frequency of nightmares compared to those who did not report such an experience. Although this result aligns with the literature on trauma and nightmares (16), our results add to the latter by showing that even the experience of common and recent troubling events can significantly increase the monthly frequency of nightmares in a community-based sample. Moreover, the NMN (38) proposes that dreams regulate fear-infused emotions by a recombination of fearful memories with non-fearful mnestic elements into dreams. Another contribution of our study was to show that, compared to individuals who did not report a recent experience of troubling event, those who did had a higher level of negative emotions with a similar level of positive ones (see Figure 3A). This mixture of emotional intensity could reflect an attempt to down-regulate negative emotions and memories through dreaming. Indeed, maintaining a “normal level” of positive emotions in the presence of an increased level of negative affects could dilute the latter within the global emotional tone of dreams, allowing their down-regulation as proposed by Perlis and Nielsen (73) and Menz et al. (74). Thus, our findings seem coherent and add to the literature on the NMN and the emotional regulation theory of dream (75).

Both the CH (11) and TST (33, 76) predict that the exposition to adverse life events would lead to more dysphoric characteristics in dreams, such as a higher frequency of nightmares, higher level of oneiric threats, and higher level of negative dream emotions. Thus, the increased frequency of nightmares and higher level of negative oneiric emotions in those who experienced a recent troubling event lends some support to both theories, but the fact that such afflicting events did not affect the threatening tone of dreams does not. Additionally, when specifically looking at dreams containing threats, the only significant associations was found for individuals having experienced a moderately severe troubling event who were more likely to experience a dream whose maximal threat severity was of moderate intensity. This suggests that, following moderately severe troubling events, the dream-production system would generate dreams with a symmetrical level of threat severity between waking and oneiric experiences. This is consistent with the TST proposing that the intensity of the oneiric threat simulations should be proportional to the magnitude of personal threat experienced during waking-life events (33). However, the fact that this association was only found for moderately, and not highly, severe troubling events does not completely align with the TST. Indeed, highly adverse experiences are postulated to be most relevant for the activation of the TSS, entailing more severe and persistent threatening dreams. This finding regarding the association between moderately threatening troubling events and moderately threatening oneiric threats also partly supports the CH due to the continuity between waking-life experiences and dream experiences. However, according to this theory, we would have expected to observe similar patterns for the other troubling events' severities, which was not the case. However, as proposed by the CH (11), the incorporation rate of waking-life experiences into dreams may depend on the time interval between both. Because the troubling event reported by our participants could have happened anytime within the last year preceding the dream report, this varying delay might have limited the degree of continuity between the waking-life troubling events and dream experiences in our study. Overall, the effects of recent troubling events on the distressing character of dream experiences seem best explained by the NMN, and offer mixed support for the TST and CH.

### Effects of Age on Dream Characteristics

No theories of dreaming have specifically addressed the ontogenetic patterns of dream experiences. For this reason, our results regarding the effects of age on dream features will be interpreted in relation to previous relevant findings. Some evidence supports a progressive declining pattern of the disturbing character of dreams across life. For instance, a linear decrease from adolescence to older age was reported for the frequency of nightmares (45), which is consistent with a previous study comparing young and older adults (46). This is congruent with our findings such that young adults reported a higher frequency of nightmares compared to older adults (>40 years old). Furthermore, our group (64, 65) previously investigated the ontogenetic patterns of several components of dream content, such as the characters, interactions, activities, and emotions of both men and women. One consistent result was the significant decrease across the lifespan of the frequency of aggressive interactions in dreams. Similarly, a study found that younger participants reported a higher frequency of dream threats, although in their study few participants were older than 40 years old (*n* = 11) (18). Together, these findings suggest a possible decrease in the frequency of oneiric threats with advancing age. Consistent with this hypothesis, we observed that adolescents and young adults reported dreams containing a higher level of oneiric threats compared to older adults (>40 years old).

Collectively, our findings suggest that the disturbing character of dreams, at the occurrence- and content-level, seem to deplete with aging. One could nonetheless speculate that the reduction in dream recall observed with advancing age (77) might explain the lower frequency of nightmares of older adults. However, it would not explain the previous and current findings of the less threatening nature of dreams in the older age groups. As it was recently proposed that dream mentation could mirror neurocognitive development across the lifespan (78, 79), one could hypothesize that such developmental processes might influence the content of dreams at different levels. For example, numerous changes pertaining to the perception of emotions, emotional processing, and regulation are incurred with advancing age. These might result from changes in the trajectories of personality traits (80), structural and functional brain changes (55, 60, 63), and coping strategies (81, 82), to name a few. Future studies will thus be required to investigate whether these developmental changes in the processing of emotion-related stimuli might relate to the declining experience of dysphoric dreams with aging. Such inquiries can bring valuable insights to theories of dreaming, shedding light on the possible mechanisms underlying their formation and function.

### Limitations and Future Directions

The main limitation of our study is the fact that the participants were not required to report the date of when the troubling experience took place in the preceding year. Therefore, it was not possible to control for the potential acute effect of the recency of the adverse experience on dream features. Future studies could thus specifically assess how the level of recency of troubling experiences progressively influences subsequent dreams' dysphoric nature and the temporal sources of these dreams (32). Additionally, we did not score the troubling events according to their nature. Instead, they were scored based on their severity as a threatening experience. Thus, we cannot determine whether the troubling events' nature could have modulated the effects observed in our study. Future studies should consider the nature of different common adverse life events and determine whether it influences the dysphoric characteristics of dreams. Another limitation is that we did not assess participant's personality traits, which may have influenced the perception of dream threats, their severity, and the negative affect elicited by them. Given that the experience of a troubling event was self-reported, individuals with a dispositional susceptibility to emotional reactivity and distress might have been more prone to report both a troubling life event and more distressing dreams (83). Indeed, it was previously shown that trait-like factors were associated with the frequency of nightmares (84, 85), oneiric threats (48) and the emotional tone (86) of dreams. Future research should incorporate questionnaires assessing personality traits and test their influence on the relationship between adverse life events and dreams. Finally, as we were interested in the potential interaction between age and the experience of a troubling personal event, and to maximize statistical power, we did not focus on the effect of gender on our results. Although previous research highlighted an impact of gender on dream characteristics (66, 87), we minimized this potential confounding effect by gender-matching our groups.

## Conclusion

Our findings lend partial support to the CH and TST but seem to favor the NMN with respect to dream formation. One exciting result is the observation of a specific mixture of oneiric emotional intensities in individuals having experienced a troubling personal event within the past year. This mixture, composed of a heightened level of negative emotions with a “normal” level of positive emotions, could serve an adaptive emotion regulation function. Future studies should investigate whether such an emotional mixture in dreams would predict better outcomes for this population's following morning mood. Furthermore, our findings may have implications beyond the understanding of dream formation to the mental health and developmental fields. Indeed, we highlighted the impact of experiencing common and recent troubling events on oneiric negative emotions and the monthly occurrence of nightmares. The recurrence of nightmares can induce waking-life distress during the following day (3). However, some evidence suggests that lucid dreaming techniques might serve as a potential intervention to reduce nightmares' occurrence (88), representing a promising avenue for future studies aiming to treat nightmare disorders. We also validated emerging evidence suggesting a decline of the disturbing character of dream experiences accompanying the advancement in age. We propose that such decline could depict the well-detailed changes in the processing of emotion-related stimuli across the human lifespan. Future studies are thus needed to further explore the potential implications of these findings for psychological adaptation in the context of adverse life events and development.

## Data Availability Statement

The raw data supporting the conclusions of this article will be made available by the authors, without undue reservation.

## Ethics Statement

The studies involving human participants were reviewed and approved by the Ethics Committee of the University of Ottawa. Written informed consent to participate in this study was provided by the participants' legal guardian/next of kin.

## Author Contributions

AL, JD, KB, and HB all contributed to the conception and design of the study. AL, HB, and EC carried out the analyses of the dreams. HB wrote an Honors Thesis in French on the basis of part of this study and collated the data in preparation of the statistical analyses. KB, AL, and HB carried out the statistical analyses. AL, KB, and JD prepared the final manuscript. JD obtained the funding for the study and acted as mentor the work. All authors contributed to the article and approved the submitted version.

## Funding

This study was supported by a grant from the Social Sciences and Humanities Research Council of Canada.

## Conflict of Interest

The authors declare that the research was conducted in the absence of any commercial or financial relationships that could be construed as a potential conflict of interest.

## Publisher's Note

All claims expressed in this article are solely those of the authors and do not necessarily represent those of their affiliated organizations, or those of the publisher, the editors and the reviewers. Any product that may be evaluated in this article, or claim that may be made by its manufacturer, is not guaranteed or endorsed by the publisher.
